# Tumor cell dormancy

**DOI:** 10.1016/j.molonc.2016.09.009

**Published:** 2017-01-03

**Authors:** Roger R. Gomis, Sylwia Gawrzak

**Affiliations:** ^1^ Oncology Program Institute for Research in Biomedicine (IRB Barcelona) The Barcelona Institute of Science and Technology Spain; ^2^ ICREA Institució Catalana de Recerca i Estudis Avançats Barcelona Spain

**Keywords:** breast cancer, cancer, dormancy, latency, metastasis

## Abstract

Metastasis is the primary cause of death in cancer patients and current treatments fail to provide durable responses. Efforts to treat metastatic disease are hindered by the fact that metastatic cells often remain dormant for prolonged intervals of years, or even decades. Tumor dormancy reflects the capability of disseminated tumor cells (DTCs), or micrometastases, to evade treatment and remain at low numbers after primary tumor resection. Unfortunately, dormant cells will eventually produce overt metastasis. Innovations are needed to understand metastatic dormancy and improve cancer detection and treatment. Currently, few models exist that faithfully recapitulate metastatic dormancy and metastasis to clinically relevant tissues, such as the bone. Herein, we discuss recent advances describing genetic cell‐autonomous and systemic or local changes in the microenvironment that have been shown to endow DTCs with properties to survive and eventually colonize distant organs.

**Abbreviations**
BMPs: bone morphogenetic proteins
CSF‐1: colony‐stimulating factor 1
CTCs: circulating tumor cells
CXCR4: C‐X‐C chemokine receptor 4
DTCs: disseminated tumor cells
ECM: extracellular matrix
EGF: epidermal growth factor
EMT: epithelial‐to‐mesenchymal transition
ER: estrogen receptor
GAS6: growth arrest‐specific 6
HSCs: hematopoietic stem cells
IGFs: insulin‐like growth factors
IL: interleukin
MET: mesenchymal‐to‐epithelial transition
MMP: matrix metalloproteinases
*NDGR1*: N‐myc downstream‐regulated gene 1
NK: natural killer
OPG: osteoprotegerin
PTHLH: parathyroid hormone‐like hormone
PTHrP: parathyroid hormone‐like protein
TGFβ: transforming growth‐factor beta
UPR: unfolded protein response
VEGFR1: vascular endothelial growth factor receptor 1

## Introduction

Despite advances in clinical oncology and basic cancer research, metastasis continues to be a lethal hallmark of cancer. In this process, malignant cells spread from the primary tumor to distant sites, where they resist conventional treatments, proliferate, and cause failure of a vital organ. Systemic dissection of the molecular, cellular, genetic, and clinical mechanisms underlying metastatic progression may lead to the development of new diagnostic and therapeutic strategies to prevent and treat metastases. However, there are some factors that challenge metastasis research, which include the biological heterogeneity of cancer types, clonal heterogeneity of primary tumors, genetic heterogeneity of cancer cells in the primary and secondary sites, and complex interactions between cancer cells and the microenvironment. In line with this, cancer types show distinct metastatic organ tropism. In addition, although steps in the metastatic cascade are part of a continuous biological sequence, their acquisition may vary from one tumor type to another. The classical simplification of metastasis into an orderly sequence of basic steps–local invasion, intravasation, survival in circulation, extravasation, and colonization–has helped to rationalize the complex set of biological properties required for a particular malignancy to progress towards overt metastatic disease (Gupta and Massague, 2006). Moreover, a progress in understanding the kinetics of the metastasis has been made in the past decade. This review focuses on the current knowledge of cancer dormancy, in particular the molecular mechanisms governing this state. The slow progression of certain subtypes of cancer under the distinct selective conditions present in various tissues gives rise to metastatic speciation. This speciation is reflected by the distinct kinetics of cancer relapse to different sites in the same patient and by the coexistence of malignant cells that differ in organ tropism in patient‐derived samples (Bos *et al*., 2009; Gupta *et al*., 2007; Lu *et al*., 2009, 2011; Padua *et al*., 2008; Zhang *et al*., 2009).

After removal of the primary tumor, metastasis may occur after a long period marked by the absence of clinical symptoms. Tumor dormancy reveals the capacity of circulating tumor cells (CTCs), disseminated tumor cells (DTCs) and/or micrometastases to remain at low numbers after primary tumor resection. These cells go undetected for long periods–sometimes years or even decades–and may explain prolonged asymptomatic residual disease and treatment resistance. Unfortunately, dormant cells will eventually produce overt metastasis, thus causing a fatal condition. As we start to unveil more about the biology of cancer cells, we can begin to address how best to treat asymptomatic residual disease. Bone metastasis‐targeted treatments represent a major advance in our understanding of tissue‐specific metastatic mechanisms and their potential use in prevention opens up new clinical avenues. However, key to determining whether dormant solitary cells or micrometastases represent valid targets is knowledge of the underlying biology of dormancy and the probability of cells progressing to active metastatic growth. This progression is poorly understood in preclinical models and even less so in the clinical context. Only through the combination and integration of cancer genetics, cell biology, cell signaling, mouse models of cancer, and cellular metastatic functions we will be able to address the following questions: What are the unique requirements of dormant metastatic cancer cells? How can we use this knowledge to improve current therapies? When these therapies shall be delivered to effectively tackle the disease? All these questions are discussed herein.

## Dormancy in the temporal course of metastasis

**Figure 1:**
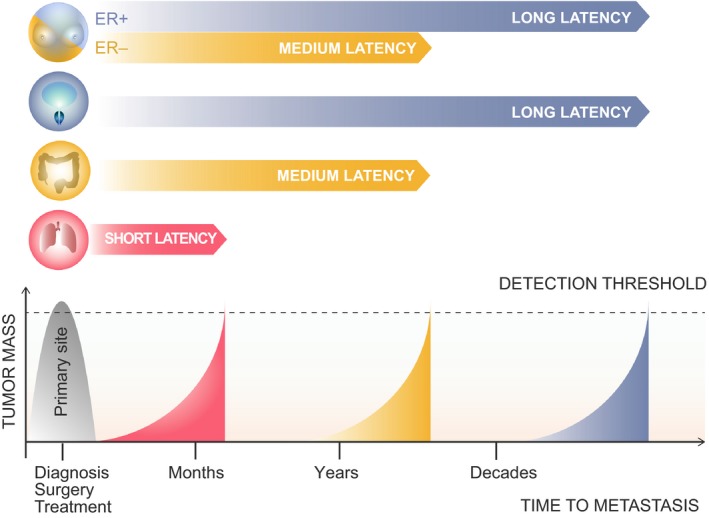
The temporal course of cancer metastasis. Metastatic relapse may occur within months, years or decades after primary tumor diagnosis, removal, and systemic treatment. Different cancer types exhibit variability in length of the latency: short for lung cancer (red), middle for colon cancer and ER− breast cancer (yellow), and long for prostate cancer and ER+ breast cancer (blue). Dashed line indicates threshold of detection symptomatic metastases.

Although the steps of the metastatic cascade are, to certain extent, uniform for most types of carcinoma, the kinetics of metastasis are highly dependent on the tumor type. Clinically detectable distant metastasis can occur simultaneously with primary tumor diagnosis or within a time ranging from weeks to decades (Fig. 1). The period between primary tumor detection and metastatic relapse is often defined as latency.

The duration of metastatic latency varies between cancer types, and for the most aggressive ones it is very short, resulting in high relapse and mortality rates following diagnosis. In lung cancer, the metastatic latency interval usually lasts only a few weeks, thus 5‐year survival rates are estimated to be around 17% (Howlader *et al*., 2016). The relapse rate is lower, reaching 30–40% in stage I lung adenocarcinoma patients (Nesbitt *et al*., 1995). In this type of cancer, malignant cells acquire metastatic traits for rapid and massive cell dissemination, followed by colonization of multiple secondary organs. Sequential metastasis to liver and lungs is often observed in colorectal cancer progression, and more than 85% of recurrences are detected within the first 3 years of follow‐up in advanced tumors (Nguyen *et al*., 2009). Therefore, this particular type of cancer shows medium latency and aggressiveness, resulting in a 5‐year survival rate of 65% (Howlader *et al*., 2016). A well‐known example of a tumor type with very long latency is prostate cancer. According to statistics from the National Cancer Institute, nearly 100% of diagnosed patients survive 5 years, and 82% are still alive 15 years after diagnosis (the most recent statistics report a 15‐year survival rate of 94% for patients diagnosed after 1994, regardless of the stage) (Howlader *et al*., 2016). The short latency in lung cancer implies that malignant cells in the primary tumor acquire most of the metastatic traits, thus enabling them to overtake organs immediately after infiltration. However, in long latent metastasis, early seeded CTCs and DTCs need time to alter or unleash the functions required for tumor initiation and expansion in the secondary site. In this case, the microenvironment of the host organ plays a key role in the acquisition of these functions (Obenauf and Massague, 2015).

**Figure 2:**
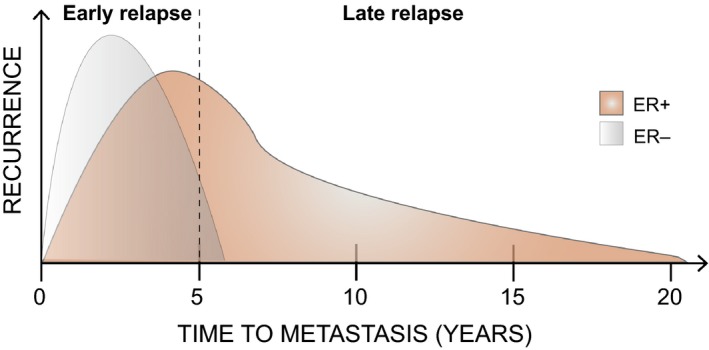
The temporal course of breast cancer metastasis. ER− breast cancer subtypes metastases typically occur within 5 years after primary tumor diagnosis (grey) whereas ER+ can relapse early (before 5 years) or late, up to decades after initial diagnosis (orange). Dashed line indicates clinical threshold for early and late relapse.

In contrast to the other types of carcinoma discussed above, breast cancer can be classified as both a medium and long latent disease (Fig. 1). Metastasis in breast cancer usually manifests asynchronously with the primary tumor and shows variable time to become clinically detected. This lag depends on the volume, stage, and molecular subtype of the primary tumor. In addition to these factors, estrogen receptor (ER) status is also related to time to recurrence (Fig. 2). ER− tumors are characterized by a more aggressive spread, thus recurrence peaks at around 2 years after diagnosis. However, the relapse rate diminishes to a low level 5 years after diagnosis. Therefore ER− subtypes are classified as either short or medium latent cancer types (Early Breast Cancer Trialists’ Collaborative, 2005; Hess *et al*., 2003; Zhang *et al*., 2013). In contrast, the ER+ sub‐type has a lower risk of recurrence than the former in the initial 5 years after diagnosis, but has a greater chronic annual risk of recurrence thereafter. Thus, more than half of the metastases of ER+ tumors occur 5 years or longer after diagnosis and surgical removal of the primary tumor. Moreover, some patients suffer recurrence after more than 20 years (Early Breast Cancer Trialists’ Collaborative Group, 2005; Hess *et al*., 2003). In addition, ER+ subtypes show higher rates of heterogeneity during the course of metastasis. In this regard, some patients will develop metastasis shortly after diagnosis and others after long latency. Strikingly, 15‐year recurrence and mortality rates for ER− and ER+ subtypes are similar in patients diagnosed at early stages of the disease (Goss and Chambers, 2010). Late recurrence decades after the initial diagnosis indicates a long latency in ER+ breast cancer metastatic progression. However, metastasis in some ER+ patients progresses rapidly, implying broad heterogeneity in recurrence patterns.

## The metastatic cascade and dormancy

**Figure 3:**
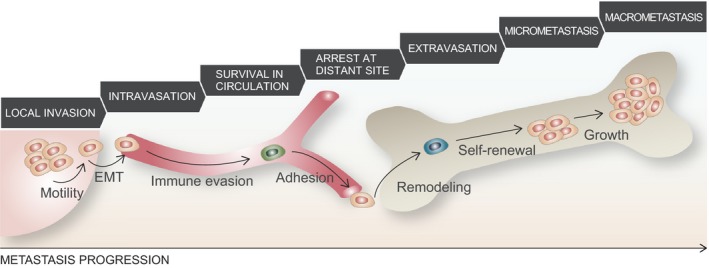
The metastatic cascade. Metastasis progresses through the sequence of steps that promote malignant cells, from primary tumor, to disseminate and colonize distant organ. Acquisition of each step is driven by specific cellular functions. Cascade steps are indicated in grey blocks, cell autonomous functions important in each step in black, circulating tumor cell in green, and disseminated tumor cell in blue.

The metastatic cascade is a series of stochastic events that collectively lead to the formation of overt metastases in a distant organ. It involves the following seven steps: invasion, intravasation, dissemination in the circulation and survival, arrest at a distant site, extravasation, tumor initiation, and, finally, outgrowth and clinical manifestation (Obenauf and Massague, 2015; Valastyan and Weinberg, 2011) (Fig. 3). Metastasis is a highly inefficient process in which each step of the cascade is a bottleneck for cancer cells and drives clonal selection. By the end of this process, only a small fraction of thousands of cells seeded daily reinitiate a tumor in a distant site. Studies based on experimental models estimate that 0.02% of melanoma cells succeed in colonizing liver after injection into a portal vain and similar results were obtained in lung colonization assay (Cameron *et al*., 2000; Luzzi *et al*., 1998). In order to metastasize, cancer cells must orchestrate diverse cellular functions to overcome the difficulties of the metastatic cascade. These functions are not only limited to cell‐autonomous traits, but also highly depend on the interaction of the metastatic cell with the tumor and host stroma. In some cases, several functions are required to implement a single step, whereas others may influence multiple ones. From a mechanistic perspective, genetic, epigenetic and translational traits alter the expression of promoter and suppressor genes, which, when combined with extended periods of dormancy, may determine metastatic latency and eventually facilitate overt clinical metastasis.

Metastasis originates in the primary tumor invasive front, where cancer cells migrate toward surrounding tissues. To achieve this movement, cell motility is altered by cytoskeleton reorganization and the secretion of extracellular matrix (ECM) remodelers, mainly proteases (Kessenbrock *et al*., 2010; Wolf *et al*., 2007). Tumor stroma composed of tumor‐associated macrophages and fibroblasts supports the invasion of metastatic cells by secreting pro‐migratory factors (Dumont *et al*., 2013; Joyce *et al*., 2004; Qian and Pollard, 2010). In order to intravasate, metastatic cells undergo an epithelial‐to‐mesenchymal transition (EMT). The loss of epithelial features, like adhesion or polarization, followed by gain of invasiveness, greatly contributes to metastasis. In this regard, the downregulation of epithelial protein E‐cadherin is a well‐established prognostic marker of metastasis (Berx and van Roy, 2009; Vleminckx *et al*., 1991). Successful intravasation also requires the formation of a new vasculature in the primary tumor by angiogenesis promoters. This neovasculature is often leaky and covered by abnormal pericytes which makes it accessible for metastatic cells (Morikawa *et al*., 2002). In addition, metastatic cells secrete factors that further increase vessel permeability, thereby facilitating their entry into the circulation. Various mediators are involved in this process, including transforming growth‐factor beta (TGFβ), and molecules produced by supportive tumor stroma, namely epidermal growth factor (EGF) and colony‐stimulating factor 1 (CSF‐1) (Giampieri *et al*., 2009; Wyckoff *et al*., 2007). The blood‐stream or lymphatic system is a hostile environment for cancer cells, and transition through vessels results in massive cell death. On the one hand, cells are challenged by innate immune natural killer (NK) cells and, on the other, they die from mechanical damage (Massague and Obenauf, 2016; Nieswandt *et al*., 1999). In order to enhance survival in the circulation, cancer cells associate with blood platelets or adhere to the endothelium at the destination site (Labelle *et al*., 2011; Valiente *et al*., 2014). After reaching the secondary site, metastatic cells are arrested in the microvasculature of the host organ prior to extravasation. Adhesion and interaction between CTCs and the host stroma facilitate microvasculature trapping (Labelle and Hynes, 2012). Extravasation in bone or liver is facilitated by extrinsic factors such as the permeability of capillaries. In other organs, such as the lungs, cancer cells acquire new functions in order to cross the vessel wall–composed of endothelial cells, basement membrane and tissue‐specific cells–and enter the parenchyma (Lawler, 2002; Padua *et al*., 2008). Vessel remodeling can be achieved by cancer cell‐secreted factors that increase the permeability of the endothelium. In the case of lung metastasis, angiopoietin‐like 4 (ANGLPTL4) disrupts cell junctions in the vascular endothelium (Padua *et al*., 2008), and parathyroid hormone‐like hormone (PTHLH) induces endothelial cell death (Urosevic *et al*., 2014).

Once metastatic cells extravasate and settle in the secondary site as DTCs, they must adapt to the microenvironment of the host organ in order to achieve homing to a distant location. Organ‐specific extrinsic factors, including stroma cells, ECM, cytokines, and growth factors, compromise the survival of DTCs. To overcome these obstacles, metastatic cells use cell‐autonomous traits that facilitate homing and survival by altering SRC tyrosine kinase signaling (Zhang *et al*., 2009) or the p38 and ERK MAPKinase signaling pathways (Adam *et al*., 2009). Similarly, they also favor stem‐cell‐like characteristics by repressing metastasis suppressor genes (Morales *et al*., 2014) and expressing the sex determining region Y‐box 2 and 9 (SOX2 and SOX9) transcription factors (Malladi *et al*., 2016; Torrano *et al*., 2016). These cells also improve homing and micrometastasis formation by creating pre‐metastatic niches at the destination. In this scenario, the primary tumor secretes systemic factors to prime tissues at the secondary site. Consequently, cells extravasate to a more permissive microenvironment. In this regard, the enzyme lysyl oxidase is a potent pre‐metastatic niche regulator (Erler *et al*., 2009). Tenascin C is another example of an ECM protein secreted by breast cancer metastatic cells to create a supportive niche in lungs (Oskarsson *et al*., 2011). Moreover, exosomes have recently been shown to promote pre‐metastatic niche formation (Peinado *et al*., 2012). Recent studies have also shown that various stroma cells, including fibroblasts, neutrophils and vascular endothelial growth factor receptor 1 (VEGFR1)‐positive bone marrow‐derived hematopoietic progenitor cells, play a central role in niche preparation (Kaplan *et al*., 2005; Malanchi *et al*., 2012; Wculek and Malanchi, 2015). Another trait improving survival of metastatic cells in a distant organ is sometimes linked to a transitory mesenchymal state of cancer cells when disseminated (Del Pozo Martin *et al*., 2015; Ocana *et al*., 2012). This mesenchymal temporarily state reverts upon reaching metastatic site, which explains why carcinoma‐derived metastases show epithelial characteristics and resemble, to certain extent, the primary tumor (Brabletz, 2012). Importantly, this process includes escape from immune responses, which are partly responsible for keeping disseminated cancer cells in check (Malladi *et al*., 2016). Whereas some lesions expand rapidly, in many tumor types DTCs are arrested and remain dormant for many years.

The last step in the metastatic cascade is the overgrowth of micrometastases into full‐blown symptomatic lesions that are clinically detectable. Metastatic cells extensively proliferate, causing failure of vital organs. This metastatic virulence is driven in an organ‐specific manner and depends on a wide range of intrinsic and extrinsic mechanisms [which have been recently reviewed elsewhere (Obenauf and Massague, 2015)].

## Minimal residual disease and dormancy

In metastatic latency, malignant cells that survived treatment and are neither detectable by conventional tests nor manifest symptoms contribute to minimal residual disease. Therefore, CTCs and DTCs in patients’ blood or bone marrow are direct evidence of minimal residual disease in metastatic latency and risk factors for recurrence (Aguirre‐Ghiso, 2007). Strikingly, metastatic cells from minimal residual disease can be transferred through organ transplants. Organs from donors diagnosed with melanoma, but successfully treated and clinically disease‐free for over 10 years, develop metastases after transplantation (Stephens *et al*., 2000; Strauss and Thomas, 2010). The isolation of CTCs from the blood of cancer patients offers valuable information about disease progression and treatment design. CTCs can be isolated in most epithelial cancers, where they represent a surrogate marker of tumor cells in transit through circulation (Alix‐Panabieres and Pantel, 2013; Yu *et al*., 2011). The results of 300 clinical trials have revealed the prognostic relevance of CTC counts with respect to metastatic progression. In addition to the counts, the analysis of surface markers expressed by CTCs can be used to monitor response to therapy and treatment‐driven clonal selection (Mitra *et al*., 2015). CTCs benefit from signals that attenuate the apoptotic outcome in circulation such as the mesenchymal transformation, stromal‐derived factors, or interepithelial cell junctions (Duda *et al*., 2010; Mani *et al*., 2008; Yu *et al*., 2012). Recent work suggests that CTC clusters derived from oligoclonal clusters of primary tumor cells are rare but represent a metastasis‐competent subset of CTCs when compared with single CTCs in a process that is dependent on the expression of Plakoglobin (Aceto *et al*., 2014).

At the cellular level, latency is often considered as dormancy. Dormancy is not a unique feature of cancer cells. Indeed, periods of dormancy and activation are essential for the self‐renewal capacities of some adult stem cells including hematopoietic stem cells (HSCs) (Trumpp *et al*., 2010). Dormant HSCs reside in the niches within the cavities of trabecular bone almost ultimately in the G0 phase of the cell cycle. Satellite cells in muscle, another example of adult stem cells, proliferate and differentiate upon activation, otherwise they are dormant (Tierney and Sacco, 2016). To a certain extent, dormant properties of metastatic cells may be attributed to the expression of tissue‐specific stem cell gene signature as was shown in studies on human metastatic breast cancer cells (Lawson *et al*., 2015a,b). However, the link between stem cell properties and cancer is part of an intense debate out of the scope of this review. In the context of metastatic dissemination and colonization, cancer cells that enter a state of dormancy are inactive in the proliferation, whereas the size of the dormant micrometastatic lesion is unchanged for a period of time. Therefore, dormancy is a crucial trait that allows DTCs and micrometastases to survive, adapt, and colonize a distant organ in the interval of long‐latent metastatic progression (Nguyen *et al*., 2009). The eventual colonization of these organs by temporarily latent DTCs involves the loss of dormant metastasis‐enforcing genes (Sosa *et al*., 2014) or, alternatively, the gain of functions that cause growth at the new metastatic site (Obenauf and Massague, 2015). Evidence from genetic metastatic signatures suggests that genes whose expression is lost in metastatic populations are the largest group of differentially expressed genes compared to primary tumors, thus suggesting that they make a significant contribution to the process (Bos *et al*., 2009; Kang *et al*., 2003; Minn *et al*., 2005). Various hypotheses might explain such an observation. Metastatic functions emerge not as a consequence of factors promoting metastasis, but rather as a result of a loss of factors supporting differentiation pathways (Casanova, 2012; Sparmann and van Lohuizen, 2006). The seeding and establishment of micrometastases requires the loss of epithelial differentiation features or gain of EMT genes (Celia‐Terrassa *et al*., 2012). These processes are transiently acquired during the early steps of the metastatic cascade and they endow DTCs with a more plastic phenotype, which may support migration, invasion, homing and initiation. It is clinically well established that breast cancer metastases show an epithelial differentiated phenotype. This paradox has been explained, in part, by the mesenchymal‐to‐epithelial transition (MET) which may contribute to awakening cancer cells and colonization of a distant organ (Ocana *et al*., 2012). Both differentiated and cancer stem cell‐like cells show slow proliferation or quiescent behaviors that dormant metastatic cells turn to their advantage under stress conditions such as chemotherapy regimens. Alternatively, the capacity of metastatic dormant cells to evade clearance by the immune response may also be central to ensuring overt colonization. Genetic or epigenetic alterations in the DTC population, systemic or local changes in the microenvironment, or a combination of these factors might eventually endow surviving DTCs with full competence for aggressive colonization.

In this context, the oncogenic background and the microenvironment may have important roles in inducing metastatic latency. Recent findings suggest that a microenvironment supportive of or restrictive for the regulation of dormancy phenotypes is crucial to provide stress signaling, autophagy, stem cell, immune and vascular niches (Sosa *et al*., 2014). For example, in the PyMT mouse model, tumor cells lacking β1 integrins fail to sense fibronectin as an environmental cue, resulting in growth arrest (White *et al*., 2004). Strikingly, DTCs are found in MMTV‐ERBB2 mice, but these animals do not develop bone metastasis. However, when DTCs are transplanted into lethally irradiated wild‐type siblings, ERBB2 +  bone marrow transplant recipients develop bone marrow carcinosis. This observation thus suggests that signals encoded in specific microenvironments govern DTC fate (Sequeira *et al*., 2007).

## Dormancy mechanisms in metastatic cancer progression

**Figure 4:**
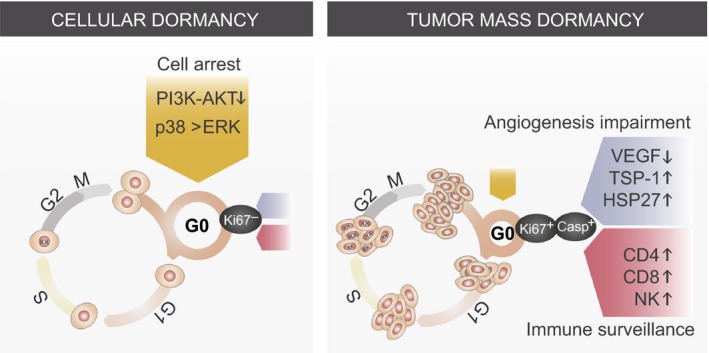
Mechanisms of cancer dormancy. Metastatic dormancy is induced and maintained by cellular (yellow), angiogenic (blue) and immune (red) mechanisms, which contribute to dormancy in different proportions. Solitary cell dormancy (cellular dormancy), defined as arrest in the cell cycle, is mediated by different signaling pathways including PI3K‐AKT low signaling and high p38 over ERK activity. In tumor mass dormancy (right) proliferation is balanced by cell death due to lack of blood supply and immune surveillance. Ki67^−^ indicates an arrested cell, Ki67^+^ states for a proliferating cell, Casp+ depicts an apoptotic cell.

Broadly defined, tumor dormancy is an arrest in tumor growth, which may occur during the formation of primary tumors or after dissemination to distant organs. However, primary tumor dormancy and metastatic dormancy appear to be distinct processes. The latter is often explained as a result of delayed adaption of DTCs to new microenvironments (Giancotti, 2013). Several distinct mechanisms have been pro posed to maintain single cell dormancy and dormant micrometastasis, including cellular, angiogenic and immunological processes. All of these contribute to the dormant period and involve various factors, such as genetic traits, tumor microenvironment components, and cancer therapeutics (Osisami and Keller, 2013) (Fig. 4).

### Solitary cell dormancy

During metastatic dormancy, a single DTC can undergo growth arrest, which is called cellular dormancy or solitary cell dormancy. In contrast, the expansion of a dividing tumor cell population in micrometastatic lesions is antagonized by a process termed tumor mass dormancy. Cellular dormancy occurs when a DTC enters a state of quiescence accompanied by decreased expression of proliferation marker Ki67. In contrast to mostly irreversible senescence, G0/G1 cell cycle arrest in the quiescent phase is likely to be responsible for cellular dormancy, hence cells are able to leave a dormant state and proliferation is re‐activated. Cell cycle arrest can be induced in response to mitogens, stress factors or other factors present in the host organ microenvironment (Osisami and Keller, 2013). To ensure their survival in the arrest phase, DTCs alter signaling pathways that coordinate metabolic homeostasis. The inhibition of the PI3K‐AKT pathway is correlated with the dormancy phenotype in DTC‐derived cell lines from breast cancer patients (Balz *et al*., 2012) and dormant head and neck squamous cell carcinoma cell lines (Schewe and Aguirre‐Ghiso, 2008).

Stress signals stemming the foreign microenvironment have also been proposed to induce dormancy in DTCs. Although reduced mitogenic signaling can trigger quiescence, specific kinases, such as dual specificity tyrosine phosphorylation‐regulated kinase 1B (DYRK1B), can trigger this stage (Deng *et al*., 2009; Jin *et al*., 2009). In pancreatic and ovarian cancer cells, DYRK1B blocks G0/G1/S transition machinery proteins, including cyclin D1, CDK4 and p27 (Deng *et al*., 2009; Ewton *et al*., 2011). Similarly, mitogen‐activated kinases such as MKK4 (MAPKK4), can induce dormancy in prostate and ovarian cancer cells by activation of JNK pathway (Hickson *et al*., 2006; Vander Griend *et al*., 2005). In a spontaneous prostate cancer metastasis model, MKK7 suppress cancer cells formation of lung metastases by inhibiting the ability of disseminated cells to colonize the distant tissue. Factors secreted by the microenvironment, such as mesenchymal cell‐derived bone morphogenetic proteins (BMPs) and growth arrest‐specific 6 (GAS6) produced by osteoblasts can directly inhibit DTC proliferation (Kobayashi *et al*., 2011; Shiozawa *et al*., 2010). In prostate cancer bone metastasis, the secretion of BMP7 activates the metastasis suppressor gene N‐myc downstream‐regulated gene 1 (*NDGR1*), thereby inducing dormancy. This subsequently leads to an increase in mitogen‐activated protein kinase p38 (p38 MAPK) activation, cell cycle inhibitor p21 expression, and cell cycle arrest (Kobayashi *et al*., 2011). Leukemia and prostate cancer cells often reside in the bone marrow and they are therefore sensitive to GAS6‐driven dormancy (Shiozawa *et al*., 2010). Breast cancer cells also bypass BMP‐mediated dormancy by expressing Coco, a BMP ligand antagonist that induces lung‐specific colonization (Gao *et al*., 2012). Recently, latent competent human breast and lung carcinoma cells have been proposed to express stem‐cell‐like SOX transcription factors, which–through the expression of WNT inhibitor DKK1 (dickkopf WNT signaling pathway inhibitor 1)–self‐impose a slow proliferating state (Malladi *et al*., 2016). Cross‐talk between mitogen‐ and stress‐induced signaling is also important for the induction of single cell dormancy. The extracellular signal‐regulated kinase (ERK1/2) to p38 MAPK ratio regulates the cell cycle since high levels of ERK1/2 activity favor proliferation. Upon downregulation of urokinase plasminogen activator receptor (uPAR), squamous carcinoma cells enter dormancy as a result of a higher ratio of p38 over ERK1/2. Increased p38 activity triggers the activation of the unfolded protein response (UPR), which upregulates the transcription factor ATF6, thus promoting cell arrest and survival (Aguirre‐Ghiso *et al*., 2001, 2003). These observations support the notion that the activation of stress signaling pathways in duces a sustained state of quiescence linked to dormancy.

### Tumor mass dormancy

In contrast to single cell dormancy caused by the arrested proliferation of solitary DTCs, the expansion of micrometastatic lesions can be inhibited by similar rates of proliferation and apoptosis. To a certain extent, cell growth arrest occurs in tu mor mass dormancy; however, tumor cells in micrometastatic lesions usually divide (Fig. 4). The proliferation to apoptosis balance is caused by enforced slow proliferation, restricted blood supply or an active immune system. All processes are tightly regulated by the tumor microenvironment. The signals that sustain the dormant state are largely unknown. Similarly, it is unknown what triggers aggressive growth and how and when it is induced. Indeed, recent evidence indicates that it is the soil conditions and not the number of seeds that determine the frequency of osteolytic bone metastasis. Fluorescently labeled disseminated human breast cancer cells, MDA‐MB‐231, determined by two‐photon microscopy *ex vivo* were detected at much higher numbers than confirmed growing bone metastases in experimental models (Wang *et al*., 2015a,b). Alternatively, stromal signals such as TGFβ and BMPs also inhibit tumor initiation properties and trigger slow proliferation and quiescence. Metastasis‐initiating cells need to overcome organ‐specific anti‐metastatic signals to resume growth. In head and neck squamous cell carcinoma, TGFB2 signaling has been suggested to induce the metastasis suppressor differentially expressed in chondrocytes 2 (DEC2), which represses CDK4 and induces p27, thus leading to slow cycling and quiescence (Bragado *et al*., 2013). Interestingly, recent work on multiple myeloma points to the coexistence of a limited number of Ki67^+^ cells in the long‐lived persistent dormant population. This observation suggests that, in certain niches, dormant cells are activated to divide. This hypothesis challenges the notion that dormancy is a synchronized period of quiescence (Lawson *et al*., 2015a,b). Unfortunately, to date, little is known about the mechanisms that sustain long‐term metastatic dormancy, particularly in contexts or niches where cell proliferation coexists as part of tissue homeostasis and regeneration. Beyond the physical and intrinsic immune limitations discussed below, in order to maintain a tumor mass dormant, cell‐autonomous mechanisms self‐impose a slow cycling feature typical of highly differentiated cells. To this end, tumor cells in this context may execute major cellular reprogramming changes capable of maintaining dormancy features for extended periods; however, these may be reversible, thus allowing the acquisition of the traits required for overt metastasis.

In order to grow beyond 1–2 mm, micrometastatic lesions induce vessel formation by secreting angiogenic factors, including vascular endothelial growth factor (VEGF), which attract endothelial and immune pro‐angiogenic cells (Conejo‐Garcia *et al*., 2004; Gao *et al*., 2008; Lyden *et al*., 2001). However, tumor mass dormancy can be maintained by the high expression of angiogenic suppressors or the downregulation of pro‐angiogenic chemokines (Ghajar *et al*., 2013; Straume *et al*., 2012). A well‐known angiogenic inhibitor is thrombospondin‐1 (TSP‐1). The upregulation of this molecule in cancer leads to poor vascularization and dormancy in *in vivo* models of breast cancer, glioblastoma, osteosarcoma, and liposarcoma (Lawler, 2002). Moreover, TSP‐1 secretion by the mature endothelium induces dormancy in DTCs, thereby indicating that this factor promotes dormancy through various mechanisms (Ghajar *et al*., 2013). In addition to secreted factors, chaperons, including heat shock 27 kDa protein (HSP27), can also regulate angiogenesis directly and by inducing pro‐angiogenic factors. The downregulation of HSP27 protein expression in angiogenic human breast cancer cells triggers long‐term *in vivo* dormancy, whereas its upregulation induces exit from dormancy and increases vascular density. Furthermore, HSP27 was shown to upregulate the secretion of the angiogenic factors belonging to the VEGF family (Straume *et al*., 2012).

The third mechanism of dormancy includes the role of the immune system in the clearance of tumor cells. The capacity of the tumor cell to initiate growth at the secondary site can be stochastic owing to newly established interactions between this cell and the target microenvironment or can already be encoded in the arriving tumor cell by attenuating the signaling cascades emanating from the environment cues or by endowing the cells with the ability to bypass the natural immune response. Cancer cells develop in a co‐evolving microenvironment that suppresses immune surveillance. However, because support is not immediately available to DTCs, most of these cells die. In addition, immune surveillance systems, in particular cytotoxic T cells and natural killer (NK) cells (Eyles *et al*., 2010), may be major players in anti‐metastatic action. Immunosuppressed patients develop tumors more often than healthy individuals. In line with this, tumor formation and progression is higher in immunodeficient mice than in immunocompetent counterparts (Shankaran *et al*., 2001). An intact immune system recognizes and removes tumor cells by cytolysis performed by adaptive immune cells, mainly cytotoxic T lymphocytes. During immunoediting, low immunogenic tumor cells exist in a balance with immunological clearance. The depletion of CD4^+^ and CD8^+^ T cells in mouse models results in escape from dormancy. These results have been supported by clinical studies showing that a lower proportion of memory T cells between the CD4^+^ and CD8^+^ cell populations in the bone marrow of breast cancer patients correlate with larger tumors (Feuerer *et al*., 2001). In additional to immunosurveillance in primary tumors, the immune system also regulates DTC numbers and the size of micrometastatic lesions (Muller *et al*., 1998). The bone marrow of patients with breast cancer that contains dormant DTCs also shows high levels of several subpopulations of immune system cells, including NK cells, macrophages, and T lymphocytes (Feuerer *et al*., 2001). Therefore, the immune system recognizes these DTCs, and memory T lymphocytes migrate to the bone marrow to control metastatic spread. Indeed, depletion of these immune cell populations increases overt metastasis (Bidwell *et al*., 2012; Malladi *et al*., 2016; Smyth *et al*., 1999), and inhibition of a negative regulator and specific NK tyrosine kinase, Mer, suppresses metastasis (Paolino *et al*., 2014). NK cell activity is suppressed in patients with advanced metastatic disease. NK cell activation is tightly regulated by activating and inhibitory signals that propagate from a panel of NK cell receptors (NKRs) expressed at the cell surface. These include three families of receptor inhibitors (C94/NKG2A, KIR and LILRB1/ILT2) that recognize class I human leukocyte antigen (HLA) molecules normally expressed in all cells. The activating NKRs include CD16 and activating KIR, NKG2D and NCR(NKp30, NKp46, NKp44) (Moretta *et al*., 2006). CD16‐expressing NK cells have been proposed to mediate antibody‐dependent cellular cytotoxicity (ADCC) upon antibody‐mediated targeted therapies, whereas the inhibitory KIR‐expressing NK cell population is the most functionally competent (high levels of Granzyme B). The action of NK and T cells is regulated by tumor cells on the basis of class I HLA expression. Variations in the expression of these proteins, together with programmed death‐ligand 1 (PD‐1) ligands in DTCs, may define the fate of these cells in response to the cytotoxic action of NK and T cells. Identifying the balance of signals that affects DTC turnover and the properties required for these cells to maintain a viable state and escape the immune system would provide valuable clues for therapeutic intervention against minimal residual disease.

## Exit from dormancy

**Figure 5:**
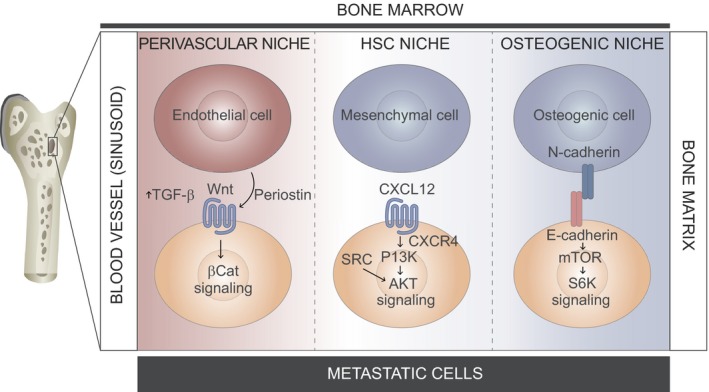
The metastatic niches in the bone marrow. In order to survive, DTC reside in different compartments in the bone marrow, so called niche. Niche occupancy depends on the pro‐survival interactions between metastatic cells (orange) and host stroma composed of different cell types of bone marrow (red or blue). In perivascular niche, sprouting neovasculature promotes micrometastatic outgrowth by secretion of periostin by endothelial cells (red) and Wnt signaling activation in metastatic cells. Mesenchymal cells in HSC niche (blue) release chemokine CXCL12 that, by CXCR4 receptor binding in metastatic cells, signals through PI3K‐AKT pathway. SRC kinase alterations in DTC amplify AKT pathway activation. DTC can also interact with osteogenic cells (blue) by formation of heterotypic adherens junctions between N‐cadherin and expressed by metastatic cells E‐cadherin. Therefore, mTOR signaling is activated promoting tumor growth.

A set of potential dormant metastasis exit mechanisms has recently been described; however, these mechanisms are strongly determined by the tissue to be colonized. Given that in long latent tumor types such as prostate and ER+ breast cancer dissemination occurs mainly in the bone, we focused on the specific mechanisms governing this process in this site (Coleman, 2001). The process of bone colonization starts by pre‐metastatic niche formation, before the arrival of metastatic CTCs. The primary tumor conditions the bone marrow by secreting soluble factors that target cells in the bone microenvironment (Weilbaecher *et al*., 2011). Molecules such heparanase, osteopontin, and lysyl oxidase facilitate the invasion, survival, and proliferation of metastatic breast cancer cells (Cox *et al*., 2015; Ibrahim *et al*., 2000; Kelly *et al*., 2005). After extravasation, metastatic DTCs may occupy various native niches in the bone, including hematopoietic stem cell, osteogenic and perivascular, to benefit from physiological signals promoting cell survival in the new environment (Fig. 5). The perivascular niche is localized around blood capillaries and, depending on the activity of endothelium, it secrets tumor‐suppressive or ‐promoting signals. DTCs localized near mature vessels are usually maintained in a quiescent state by endothelium‐derived TSP‐1, which is a potent tumor suppressor (Ghajar *et al*., 2013). As a result of neovascular sprouting, which disrupts vessel homeostasis, endothelial cells release more tumor‐promoting signals, such as ECM molecules, and growth factors, including periostin and active TGFβ, which drive micrometastatic outgrowth (Ghajar *et al*., 2013) (Fig. 5). Since the bone marrow is permissive for the homing and residence of HSCs, the HSC niche seems to be a protective environment for DTCs because it provides pro‐survival chemokines that sustain the viability of metastatic cells (Yoneda, 2000). For example, the secretion of stromal cell‐derived factor 1 (SDF1; also known as CXCL12) by mesenchymal cells in the bone marrow promotes the survival of metastatic cells that express C‐X‐C chemokine receptor 4 (CXCR4) (Kang *et al*., 2003). Moreover, overexpression of the tyrosine kinase SRC in DTCs amplifies the CXCR4‐induced activation of the PI3K‐AKT pathway (Zhang *et al*., 2009) (Fig. 5). The third niche that DTCs occupy is the osteogenic niche, where interactions with the stroma enhance mTOR activity and drive progression from single cells to micrometastases prior to osteolysis (Wang *et al*., 2015a,b) (Fig. 5). These distinct mechanisms that metastatic cells use to survive in the bone microenvironment and to exit dormancy reflect the heterogeneity of metastatic populations. Niche occupancy depends on the traits that cells acquire during the metastatic cascade, followed by the interactions between tumor and host cells. Therefore, DTCs home to the bone, start to proliferate (often after a period of dormancy), form micrometastatic lesions, and finally induce vicious cycles of bone lysis and tumor growth.

**Figure 6:**
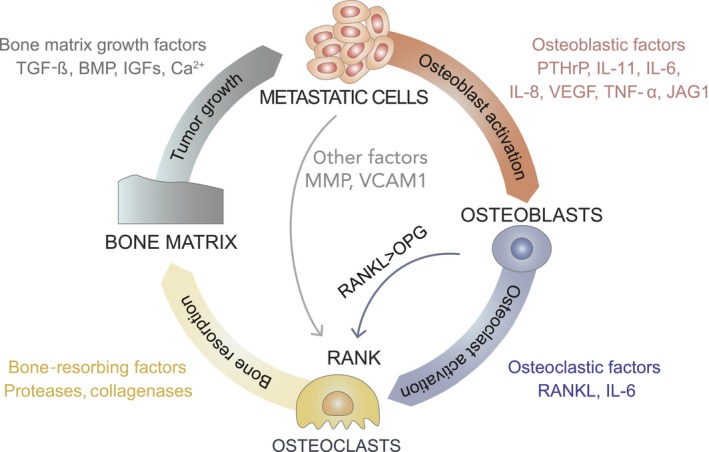
The bone metastasis vicious cycle. During the osteolytic cycle, metastatic cells in the bone microenvironment produce molecules that stimulate osteoclastic bone resorption directly by MMP and VCAM1 (grey), or indirectly through osteoblast activation by osteoblastic factors (orange). Activated osteoblasts secret osteoclastic factors (blue) mainly RANKL to promote bone degradation by proteases and collagenases (yellow). The consequence of increased resorption is the release of growth factors from the bone matrix (grey) that feed back to the metastatic cells, further stimulating their growth. Ca^2+^ that stands for calcium ions.

In the final phase of metastatic colonization, cancer cells control the bone microenvironment to activate osteoclasts and suppress bone formation. This is achieved by paracrine crosstalk among cancer cells, osteoblasts, osteoclasts, and the bone matrix (Fig. 6). Cancer cells secrete osteolytic factors that activate bone‐resorbing osteoclasts. To activate osteoblasts, metastatic cells produce cytokines and growth factors, including parathyroid hormone‐like protein (PTHrP), interleukin (IL)‐11, IL‐6, IL‐8, VEGF, and tumor necrosis factor (TNF‐a) (extensively reviewed in (Weilbaecher *et al*., 2011; Kozlow and Guise, 2005)]. As a result, osteoblasts release soluble receptor activator of nuclear factor kappa‐B ligand (RANKL) and inactivate its antagonist osteoprotegerin (OPG). The ratio of RANKL to OPG is critical for osteoclast activation since OPG prevents RANKL from binding to its receptor RANK. Once activated upon ligand binding, the multinucleated osteoclasts attach to the bone surface and release acid and proteolytic enzymes, such as cathepsin K and matrix metalloproteinases (MMP) to resorb the bone matrix. Osteolysis releases growth factors stored in the matrix, including TGFβ, insulin‐like growth factors (IGFs), and BMPs, as well as calcium ions, into the bone microenvironment. In addition to tumor growth enhancement, in metastatic cells, TGFβ activates both Smad‐dependent and Smad‐independent signal pathways to induce PTHrP (Kang *et al*., 2005; Yin *et al*., 1999). Therefore, tumor growth is stimulated, leading to the production of additional osteolytic and osteoblastic factors and resulting in the vicious cycle of bone metastasis. In addition, bone resorption can be promoted by the Notch signaling pathway, which results in IL‐6 secretion upon binding of tumor‐derived JAGGED‐1 (JAG‐1) to osteoblasts (Sethi *et al*., 2011).

## Clinical and experimental implications

The new extended treatments and increase in overall survival achieved with current therapies have highlighted the need for new diagnostic tests to identify patients at high risk of suffering late metastasis and, hence, those that could benefit most from a rational system that would apply treatments to prevent survival of disseminated tumor cells in the bone marrow. Presumably, systemic therapy delivered after tumor removal aims to prevent relapse. However, the current pharmacological arsenal used in the adjuvant setting (chemotherapy) targets growing tumor cells rather than dissemination. The systemic nature of the metastatic disease, the heterogeneity of metastatic tumors, the multitude of interconnected pathways, and the resistance mechanisms suggest a difficult pharmacological approach. Collectively, these facts imply that attention should be directed to preventing metastasis rather than treating it(Coleman, 2012a,b). Thus, focus on high‐risk patients is pivotal to effectively eliminate residual disseminated disease (Pavlovic *et al*., 2015). Unfortunately, when tested in the overall cancer population in the preventive adjuvant setting, inconclusive results have been reported for bone‐modifying drugs to date, in spite of their use in clinical practice to control bone metastasis morbidity (skeletal related events and hypercalcemia) (Coleman, 2012a,b; Smith *et al*., 2015). A better understanding of the bases of metastatic dormancy and colonization and better drugs are needed to develop improved treatments to address this unmet medical need. To this end, drugs that could prevent metastasis by targeting metastatic cell‐autonomous functions that sustain dormancy or mechanisms that support their existence in the preventive setting represent a new opportunity to eliminate minimal residual disease, thus enhancing the quality of patients’ lives.

To date, there are few experimental models available to study the latency phase of tumor growth. Particularly, the striking differences in the growth kinetics between cancer cell lines and mouse models compared to human cancer makes this studies difficult (Klein, 2009). Thus, suitable experimental models would require a latent phase that lasts up to at least 6–8 weeks. Recent studies have provided a number of models indicating mechanisms of cellular or tumor mass dormancy as an important contributors to long‐latency. Using a human ER− breast cancer cell line (Lu *et al*., 2011), a single clone population was isolated that infrequently formed overt metastases from dormant micrometastases in the bone. Also, mathematical modeling showed that patients with long‐latent breast cancer have between 1 and 5 micrometastases at 10 years post‐resection, thereby indicating that small numbers of lesions maintain dormancy (Willis *et al*., 2010). Several lines of evidence indicate that the quiescence of single cells is an important contributor to long latency. DTCs in the bone marrow of breast cancer patients are largely non‐proliferative and, in contrast to CTCs, can persist in the target organs for long periods (Klein, 2011). A recent report showed that, upon orthotropic injection, human ER− breast cancer cells disseminate to various organs, including liver, lung, brain, and bone marrow, and undergo cellular dormancy before the formation of micrometastases (Ghajar *et al*., 2013). Also, in a syngeneic mouse model of breast cancer, dormancy is governed by the quiescence of solitary cells. Single 4TO7 cells enter arrest immediately upon infiltrating the lung and are therefore unable to form micrometastatic lesions (Gao *et al*., 2012). Currently, cellular dormancy is associated mainly with solitary cells, while dormant macrometastatic lesions are considered to consist of actively proliferating cells balanced with the same number of apoptotic cells (Wells *et al*., 2013). Moreover, these two forms of dormancy were thought to be exclusive and sequential events. However, recent studies suggest that G0 cell dormancy is responsible not only for the arrest of solitary cells, but also contributes to tumor mass dormancy, thereby suggesting that a variety of mechanisms can synergistically promote long latency (Lawson *et al*., 2015a,b). Thus, mechanisms of cellular dormancy manifested as quiescence are not exclusive to solitary cells but contribute to tumor mass dormancy. It is unclear how slow cycling and quiescence are imposed in a cell‐autonomous manner, how the niche specifically contributes to such a process, and how a continuously dormant single cell or tumor mass evolves and acquires properties that support symptomatic metastasis.

## Concluding remarks

An important goal of current research is to provide new drugs to increase the overall survival of cancer patients. Although new systemic therapies and surgical improvements have had a significant impact on the field in recent years, many patients still develop metastasis. To overcome current limitations, metastasis prevention seems a far more logical option than the treatment of advanced metastatic disease. For example, mTOR inhibitors have an impact on overall survival of advanced ER+ breast metastatic cancer, but this effect is limited in time. Thus, the most plausible and challenging window opportunity for new treatments to prevent metastasis is by modifying the adjuvant setting defined by dormancy. This period reflects the capacity of disseminated tumor cells or micrometastases to persist at low numbers for long periods after tumor resection remaining as asymptomatic residual disease. However, to effectively target this process, deeper knowledge of the cancer patients at risk of distant metastasis is needed to effectively deliver proper drugs. In addition, a greater understating of the mechanisms underlying tumor dormancy and how these are overcome to allow metastasis regrow are paramount if we are to find new strategies to tackle asymptomatic residual disease.
